# A Pyroptosis-Based Prognostic Model for Immune Microenvironment Estimation of Hepatocellular Carcinoma

**DOI:** 10.1155/2022/8109771

**Published:** 2022-01-10

**Authors:** Zhihong Chen, Yiping Zou, Yuanpeng Zhang, Zhenrong Chen, Fan Wu, Haosheng Jin, Ning Shi

**Affiliations:** ^1^Department of General Surgery, Guangdong Provincial People's Hospital, Guangdong Academy of Medical Sciences, Guangzhou 510080, China; ^2^College of Medicine, Shantou University, Shantou 515041, China

## Abstract

**Background:**

Hepatocellular carcinoma (HCC), an aggressive malignant tumor, has a high incidence and unfavorable prognosis. Recently, the synergistic effect of pyroptosis in antitumor therapy and regulation of tumor immune microenvironment has made it possible to become a novel therapeutic method, but its potential mechanism still needs further exploration.

**Methods:**

Differentially expressed genes with prognostic value in Liver Hepatocellular Carcinoma Project of The Cancer Genome Atlas (TCGA-LIHC) cohort were screened and incorporated into the risk signature by Cox proportional hazards regression model and least absolute shrinkage and selection operator. Kaplan-Meier (KM) curves and receiver operating characteristic (ROC) curves were applied to conduct survival comparisons and estimate prediction ability. The dataset of Liver Cancer-RIKEN, Japan Project from International Cancer Genome Consortium (ICGC-LIRI-JP) cohort was used to verify the reliability of the signature. Correlation analysis between clinicopathological characteristics, immune infiltration, drug sensitivities, and risk scores was conducted. Functional annotation analyses were performed for the genes differentially expressed between high-risk and low-risk groups.

**Results:**

A risk signature consisting of 6 pyroptosis-related genes in HCC was developed and validated. KM curves and ROC curves revealed its considerable predictive accuracy. Higher risk scores meant more advanced grade, higher alpha-fetoprotein level, and stronger invasive ability. Overexpressed genes in high-risk population were more enriched in the immune-associated pathways, and these patients might be more sensitive to immune checkpoint inhibitors instead of Sorafenib. Intriguingly, 6 identified genes were promising to be prognostic biomarkers and therapeutic targets of HCC.

**Conclusions:**

The signature may have crucial clinical significance in predicting survival prognosis, immune infiltration, and drug efficacy based on pyroptosis-related genes.

## 1. Introduction

Primary liver cancer commonly has a high incidence and poor prognosis, with the increasing morbidity rate ranking the sixth and cancer-related mortality rate ranking the third all over the world, of which, hepatocellular carcinoma (HCC) accounts for 75%-85% [1]. Patients with a background of chronic liver diseases are more likely to develop HCC, and the main risk factors of HCC include long-term hepatitis B virus and hepatitis C virus infection, nonalcoholic fatty liver, overconsumption of alcohol, and dietary aflatoxin exposure [2]. With a high prevalence of hepatitis B, HCC becomes common cancer in China, with an incidence rate of 26.67 cases per 100,000 people, and over 373,000 cases are new cases each year [3]. The early-stage HCC can achieve considerable clinical cure by the methods like surgical resection, liver transplantation, interventional therapy, or ablation. However, diagnosed with advanced HCC, most of the patients are unable to receive radical treatment, which often leads to worse therapy efficacy, poorer survival prognosis, and shorter survival time [4].

In recent years, ferroptosis, necroptosis, and pyroptosis have been found to be associated with the tumor initiation and progression and the activation of antitumor immunity during the investigation of tumor cell death [5]. Pyroptosis, a programmed cell death featured by cell swelling, cell membrane rupture, the release of intracellular inflammatory factors, and induction of inflammatory response is considered to be tightly associated with antitumor immune effect and promote the tumor cell immunogenic death synergistically when the tumor responds to chemotherapy or radiotherapy. The activation of two main biological approaches can form perforin and induce pyroptosis, like caspase 1/4/5/11-regulated gasdermin D- (GSDMD-) dependent activation and caspase 3-regulated gasdermin E- (GSDME-) dependent activation, which can lead to the release of inflammatory mediators and strengthen the tumor-killing effect of CD8+ T cells and other functional antitumor immune cells. Cleavage of GSDMD by inflammatory caspases can form perforin on the cell membrane and is required for classical pyroptotic cell death and IL-1*β* release [6]. Chemotherapeutic drugs can activate caspase 3/GSDME to induce pyroptosis [7]. The expression level of some pyroptosis-related proteins is related to tumor aggressive biological behaviors, like the larger tumor size, the higher histopathological grade, and the more advanced tumor stage. In breast cancer, cervical cancer, colorectal cancer, and other multiple cancers, pyroptosis plays an important role in tumor promotion and inhibition [8]. Therefore, pyroptosis is considered to become the promising direction for tumor treatment. Regulation of pyroptosis may improve the therapeutic effect in tumor immunotherapy.

Recently, great progress has been made in immunotherapy of HCC, especially immune checkpoint inhibitors and even combined immunotherapy, and it is hopeful to improve the survival rate of the patients. As an important part of the comprehensive treatment of HCC, it is worth being explored. Nevertheless, not all patients can achieve the expected objective response rate after immunotherapy due to intratumoral heterogeneity. Therefore, there is an urgent need for effective strategies to identify the population that can benefit from immunotherapy [9]. Referring to the existing research findings, a close relationship between pyroptosis and antitumor immunotherapy has been reported. However, the specific function and mechanism of pyroptosis in HCC remain unclear. Hence, in the present study, the differentially expressed pyroptosis-related genes with prognostic value were analyzed, identified, and incorporated into the risk signature to assess survival prognosis, immune microenvironment, and drug sensitivity in HCC.

## 2. Materials and Methods

### 2.1. Data Acquisition and Processing

The gene expression profiles and corresponding clinical information were downloaded from Liver Hepatocellular Carcinoma Project of The Cancer Genome Atlas (TCGA-LIHC, http://cancergenome.nih.gov/) and Liver Cancer-RIKEN, Japan Project of International Cancer Genome Consortium (ICGC-LIRI-JP, https://dcc.icgc.org/). After excluding the patients without detailed survival information, 365 patients from the TCGA-LIHC cohort were enrolled in the present study. Informed consent was not compulsory because of the patients' unknown identity in the above cohorts. The pyroptosis-related gene sets include REACTOME_PYROPTOSIS obtained from the Molecular Signatures Database (MSigDB v7.4, http://www.gsea-msigdb.org/gsea/index.jsp/) and GO_BP_PYRPTOSIS downloaded from the Gene Ontology Consortium (http://geneontology.org/). The other pyroptosis-related genes were searched from the relevant literature [10, 11]. A total of 58 pyroptosis-related genes were included in the bioinformatic analysis.

### 2.2. Differential Expression Analysis of Pyroptosis-Related Genes

Differentially expressed gene analysis of pyroptosis-related genes between 374 HCC samples and 50 adjacent normal samples in the TCGA-LIHC cohort was conducted by using the limma R package, and the threshold value of false discovery rate (FDR) was set to less than 0.05.

### 2.3. Construction and Validation of a Pyroptosis-Related Gene Signature

The differentially expressed pyroptosis-related genes with significant prognostic value were identified by univariate Cox regression analysis. The correlation of the prognostic candidates was evaluated with Pearson correlation analysis. The least absolute shrinkage and selection operator (LASSO) method was utilized in the selection of the prognostic candidates and the construction of the prognostic pyroptosis-related gene signature. The formula for the individual risk score calculation was as follows: Risk Score = ∑_*i*_^*j*^*Xi*∗*Yi* (*X*: regression coefficient; *Y*: gene expression; *i*: prognostic gene). The distribution of survival status and risk score for each patient was plotted. In accordance with the median risk scores, the patients were classified into a high-risk group and a low-risk group. The survival analysis between subgroups with different risk levels was performed with the Kaplan-Meier (KM) method, and the survival outcome was compared. Receiver operating characteristic (ROC) curve was used in the estimation of the prediction ability of risk signature for the survival rate at 1, 2, and 3 years. Principal component analysis (PCA) and *t*-distributed stochastic neighbor embedding (*t*-SNE) algorithms were used in the dimension reduction analysis and the assessment of the clustering ability of risk signature. The ICGC-LIRI-JP cohort was served as the external validation cohort and repeated the same analysis process as above. The independent prognostic value of the risk signature was tested in univariate and multivariate Cox regression analyses, together with the patients' clinicopathological characteristics. GEPIA (http://gepia.cancer-pku.cn/) was a convenient online tool to conduct the survival analysis of different risk groups according to the median value of each prognostic gene expression in the TCGA-LIHC cohort [12].

### 2.4. Comparison of Clinicopathological Features between Risk Subgroups

The clinicopathological features of the high-risk group and the low-risk group, including survival status, age, gender, AJCC TNM stage, histological grade, T stage, N stage, M stage, alpha-fetoprotein (AFP) level, and vascular invasion situation, were compared by the Chi-square test. The results were shown in the form of a heat map. The risk scores of the patients with different ages, genders, AJCC TNM stages, histological grades, and T stages were compared with the methods of the Wilcoxon test and Kolmogorov–Smirnov test. M stage and N stage were not included in the comparison analysis due to the few positive cases. The results were exhibited in the form of the boxplots.

### 2.5. Comparison of Immune Activities and Drug Sensitivities between Risk Subgroups

The differentially expressed genes between high-risk and low-risk groups were extracted and analyzed by Gene Oncology (GO) term and Kyoto Encyclopedia of Genes and Genomes (KEGG) pathway analysis using the R package clusterProfiler, and threshold value was set as FDR < 0.05 and *P* value < 0.05 [13].

ESTIMATE (Estimation of STromal and Immune cells in MAlignant Tumor tissues using Expression data) was used to estimate the infiltration situation of immune cell and stromal cell in tumor tissue for each sample. The immune score and the stromal score represented the infiltration extent of immune cells and stromal cells, respectively [14]. The comparison was made between different risk groups. The score of tumor immune dysfunction and exclusion (TIDE, http://tide.dfci.harvard.edu/) could predict the patients' response to immune checkpoint inhibitors by estimating multiple published transcriptomic biomarkers according to pretreatment gene expression profiles of the tumor, and the higher TIDE score meant the lower response rate of immune checkpoint inhibitors (ICIs) [15].

Single sample gene set enrichment analysis (ssGSEA) could be used in the calculation of the enrichment score that represented the absolute enrichment degree of the specific gene set in each sample by empirical cumulative distribution function based on the given gene expression profile. ssGSEA scores derived from 16 different immune cells and 13 immune signal pathways of the high- and low-risk groups were obtained using the Gene Set Variation Analysis (GSVA) package and then compared using the limma package in R [16, 17].

The immune infiltration in tumor tissues could be estimated and evaluated by some online tools and bioinformatic algorithms and packages according to the transcriptomic profiles of the tumor tissues, like xCell (https://xcell.ucsf.edu/), TIMER (https://cistrome.shinyapps.io/timer/), EPIC (https://gfellerlab.shinyapps.io/EPIC_1-1/), CIBERSORT (https://cibersort.stanford.edu/), quanTIseq algorithm (http://icbi.at/quantiseq), and MCP-counter package [18–22]. The correlation between risk score and the extent of immune cell infiltration was calculated to explore the relationship between risk score and tumor immune microenvironment.

By using the pRRophetic package, the drug sensitivities of tumor samples could be evaluated by the 50% inhibiting concentration (IC50) derived from the given gene expression profiles [23]. IC50 refers to the drug concentration required when the number of viable cells is reduced by half after administration, which can reflect the drug therapeutic efficacy and measure the tolerance of tumor cells to the drugs. The lower IC50 indicated the higher response rate of tumor cells to drug. The IC50 of Sorafenib for high-risk and low-risk groups were calculated and compared. The gene expression level of immune checkpoints (PD-1, PD-L1, CTLA4, HAVCR2, LAG3, and TIGIT) between high- and low-risk groups was compared with the Wilcoxon test method, and their immune checkpoint blockade efficiency was evaluated through the gene expression comparison.

### 2.6. Statistical Analysis

R version 4.1.0 software (https://cran.r-project.org/) was the main tool to conduct the statistical analysis, and a *P* value of <0.05 was considered as statistically significant.

## 3. Results

### 3.1. Identification of Differentially Expressed Pyroptosis-Related Genes

**Figure 1:**
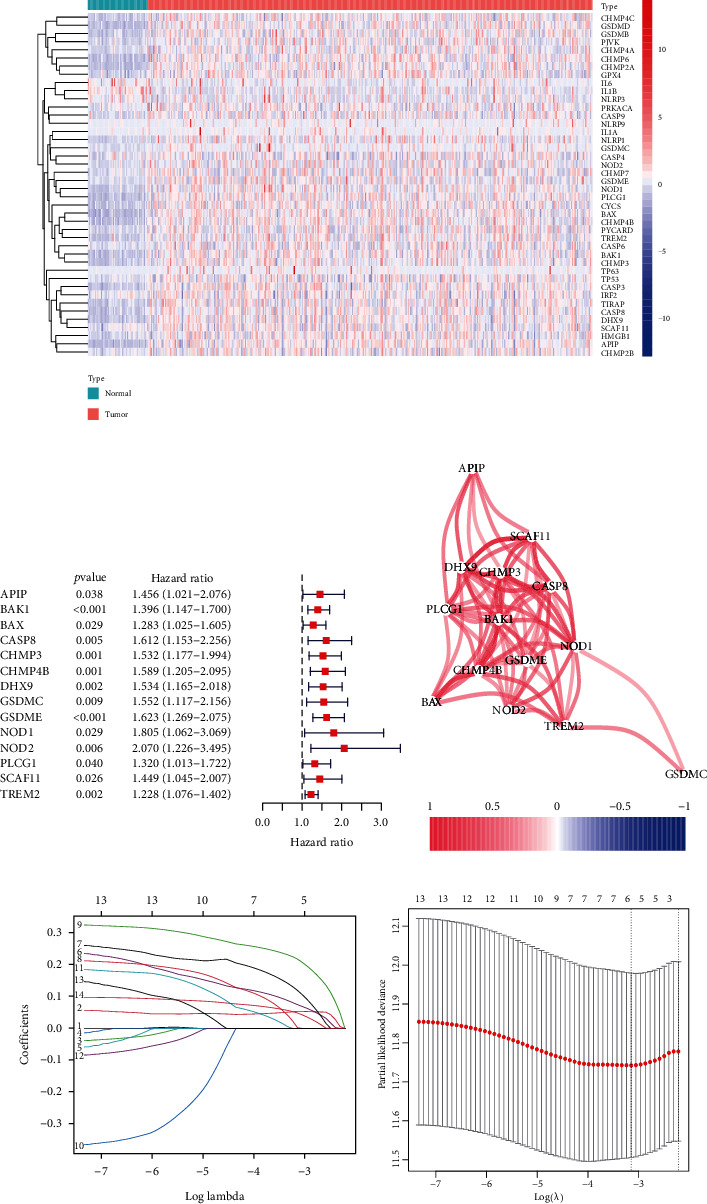
Expression, correlation, and prognostic information of pyroptosis-related genes. (a) Heat maps of 42 differentially expressed pyroptosis-related genes expressed in tumors and adjacent normal tissue. (b) Forest plot of 14 differentially expressed pyroptosis-related genes with survival prognostic value. (c) Correlation plot of 14 pyroptosis-related genes with survival prognostic value. (d, e) Least absolute shrinkage and selection operator process of pyroptosis-related genes with survival prognostic value.

A total of 58 pyroptosis-related genes were obtained from literature review and data retrieval. As was shown in Figure 1(a), there were 42 differentially expressed genes between HCC and adjacent normal tissues, comprising 39 upregulated genes and 3 downregulated genes.

### 3.2. A Prognostic Signature Based on Pyroptosis-Related Gene

**Figure 2:**
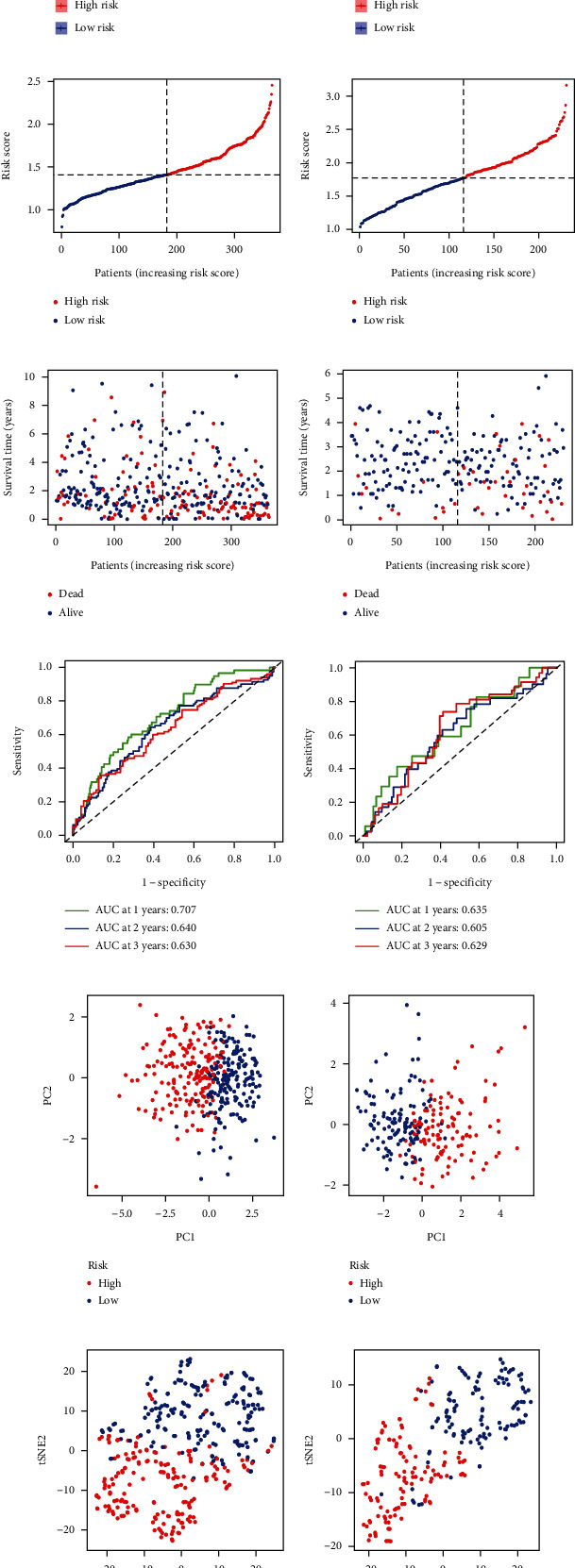
Development and validation of the risk signature in TCGA-LIHC and ICGC-LIRI-JP. (a, b) Kaplan-Meier survival plots of high-risk and low-risk groups in TCGA-LIHC and ICGC-LIRI-JP. (c–f) The distribution of risk score and survival outcome of the high-risk and low-risk groups in TCGA-LIHC and ICGC-LIRI-JP. (g, h) ROC curves of the survival models in TCGA-LIHC and ICGC-LIRI-JP. (i–l) PCA and *t*-SNE plot of the survival models in TCGA-LIHC and ICGC-LIRI-JP.

Univariate Cox regression analysis identified 14 differentially expressed pyroptosis-related genes (APIP, BAK1, BAX, CASP8, CHMP3, CHMP4B, DHX9, GSDMC, GSDME, NOD1, NOD2, PLCG1, SCAF11, and TREM2) with survival prognostic value (Figure 1(b)). Their high expressions were related to the poor prognosis of HCC (all HR > 1, *P* < 0.05). Subsequently, the results of Pearson correlation analysis indicated the positive correlation of 14 prognostic candidates (Figure 1(c)). Finally, the LASSO Cox regression screened 6 genes and incorporated them into the formula to calculate risk score: 0.052∗BAK1 expression + 0.081∗CHMP4B expression + 0.131∗DHX9 expression + 0.023∗GSDMC expression + 0.225∗GSDME expression + 0.053∗TREM2 expression (Figures 1(d) and 1(e)). In accordance with the median value of risk scores, the TCGA-LIHC cohort was divided into the high- and low-risk group. The distribution of risk scores and survival status revealed a higher risk score was associated with a higher probability of death. Survival analysis indicated that the high-risk group had a worse survival prognosis compared with the low-risk group (*P* < 0.001, Figures 2(a), 2(c), and 2(e)). The ROC curves suggested the predictive accuracy of survival prognosis at the 1, 2, and 3 years were 0.707, 0.640, and 0.630, respectively (Figure 2(g)). The results of PCA and *t*-SNE confirmed the risk signature had a considerable clustering ability (Figures 2(i) and 2(k)).

Data from the ICGC-LIRI-JP cohort were served as external verification data to validate the risk signature. In the same way, the cohort was divided into high-risk and low-risk groups according to the median risk score. Similarly, the patients with higher risk were more likely to have poorer survival outcomes (*P* < 0.001, Figures 2(b), 2(d), and 2(f)). The ROC curves suggested the predictive accuracy of survival prognosis at the 1, 2, and 3 years were 0.635, 0.605, and 0.629 (Figure 2(h)). The results of PCA and *t*-SNE also demonstrated the discrimination ability of the risk signature (Figures 2(j) and 2(l)).

**Figure 3:**
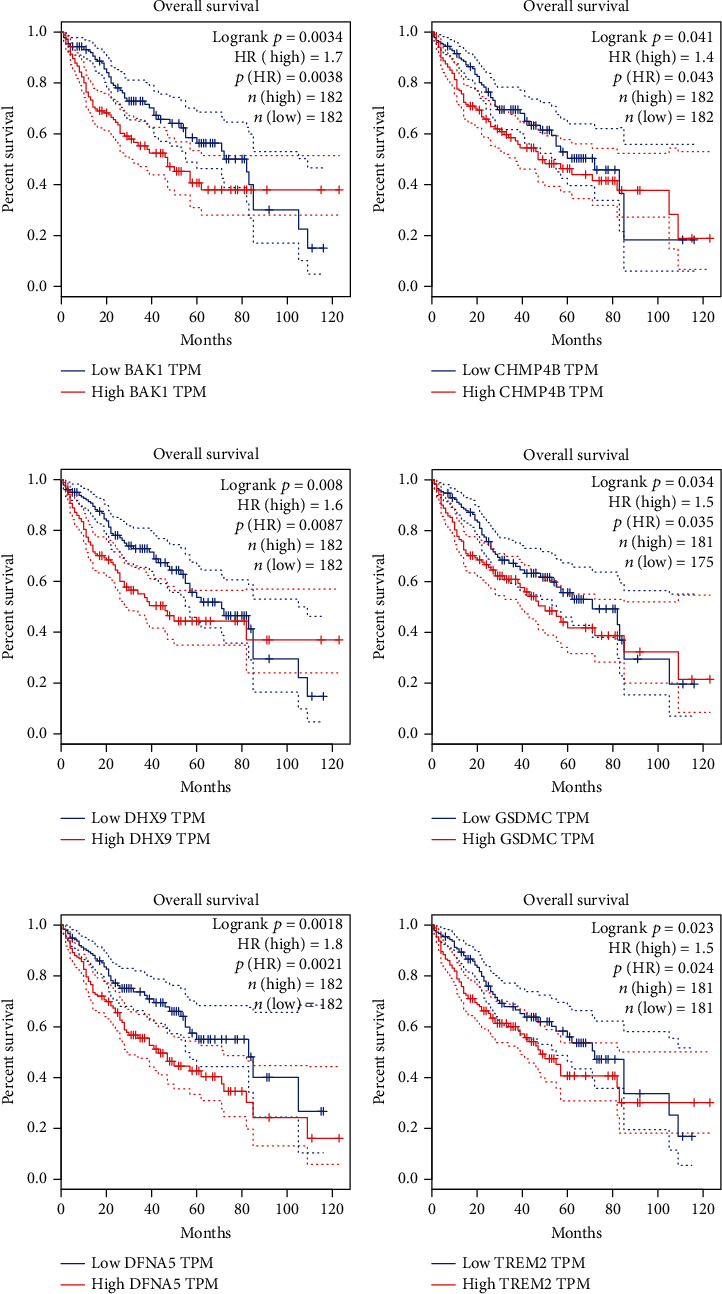
Survival prognostic analysis of the expression level of 6 determined pyroptosis-related genes in HCC: (a) BAK1, (b) CHMP4B, (c) DHX9, (d) GSDMC, (e) DFNA5 (GSDME), and (f) TREM2.

The survival analysis results of GEPIA further confirmed that the gene expression of BAK1, CHMP4B, DHX9, GSDMC, GSDME, and TREM2 had the overall survival predictive effect for the patients with HCC (all HR > 1, *P* < 0.05) and the increased expression might cause the poor survival outcome (Figures 3(a)–3(f)).

### 3.3. Clinicopathological Features, Immune Activities, and Drug Sensitivities between High- and Low-Risk Groups

**Figure 4:**
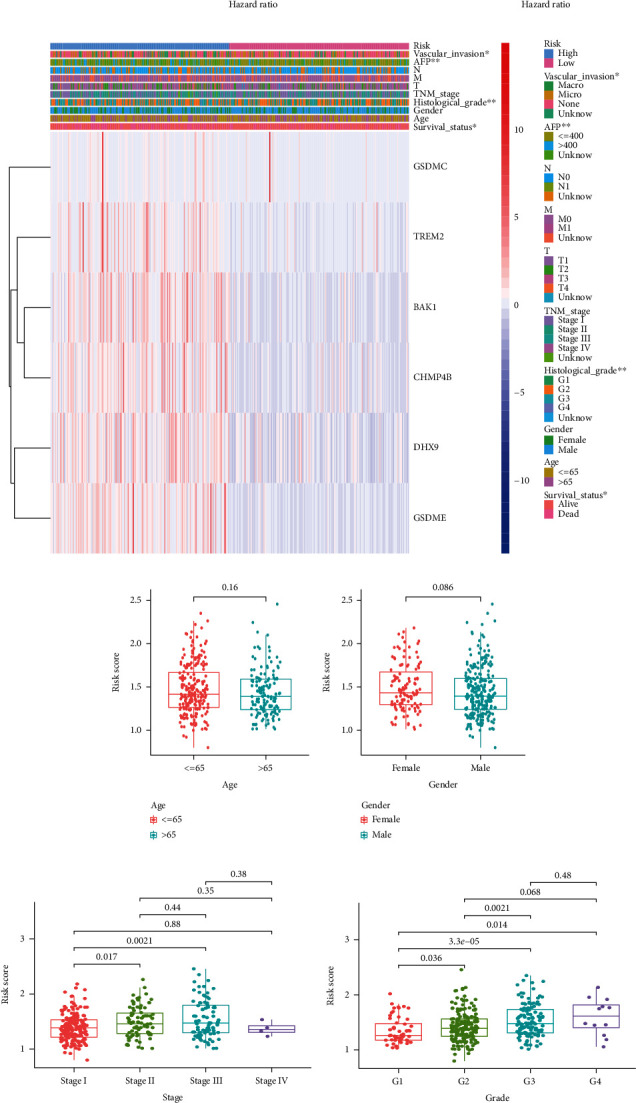
Independent analysis and subgroup comparison of risk signature and clinical factors. (a) Forest plot of univariate Cox regression analysis in TCGA. (b) Forest plot of multivariate Cox regression analysis in TCGA. (c) Heat map of 6 determined pyroptosis-related genes and clinical factors (^∗∗∗^*P* < 0.001, ^∗∗^*P* < 0.01, and ^∗^*P* < 0.05). (d–g) The subgroup comparison of risk scores in different ages, genders, AJCC TNM stages, and histological grades.

Univariate Cox regression analysis showed AJCC TNM stage (*P* < 0.001), T stage (*P* < 0.001), and risk score (*P* < 0.001) were related to the patients' survival prognosis (Figure 4(a)). Furthermore, the results of multivariate Cox analysis confirmed the independent prognostic effect of the risk score (Figure 4(b)).

The results of the Chi-square test indicated that survival status (*P* < 0.05), tumor histological grade (G1, G2, G3, and G4, *P* < 0.01), vascular invasion situation (none, microvascular, and macrovascular invasion, *P* < 0.05), and AFP level (≤400 *μ*g/L and >400 *μ*g/L, *P* < 0.01) existed significant difference between high-risk and low-risk groups (Figure 4(c)). Nevertheless, other clinicopathological features like age, gender, M stage, and N stage showed no significant difference in the patients with high risk and low risk. The risk scores in the subgroups with different clinicopathological features, like age (≤65 years old and >65 years old), gender (female and male), histological grade (G1, G2, G3, and G4), AJCC TNM stage (stage I, stage II, stage III, and stage IV), and T stage (T1, T2, T3, and T4), were compared. The boxplot illustrated that the patients with higher histological grade, advanced TNM stage, and larger tumor size tend to have higher risk scores (Figures 4(d)–4(g)).

**Figure 5:**
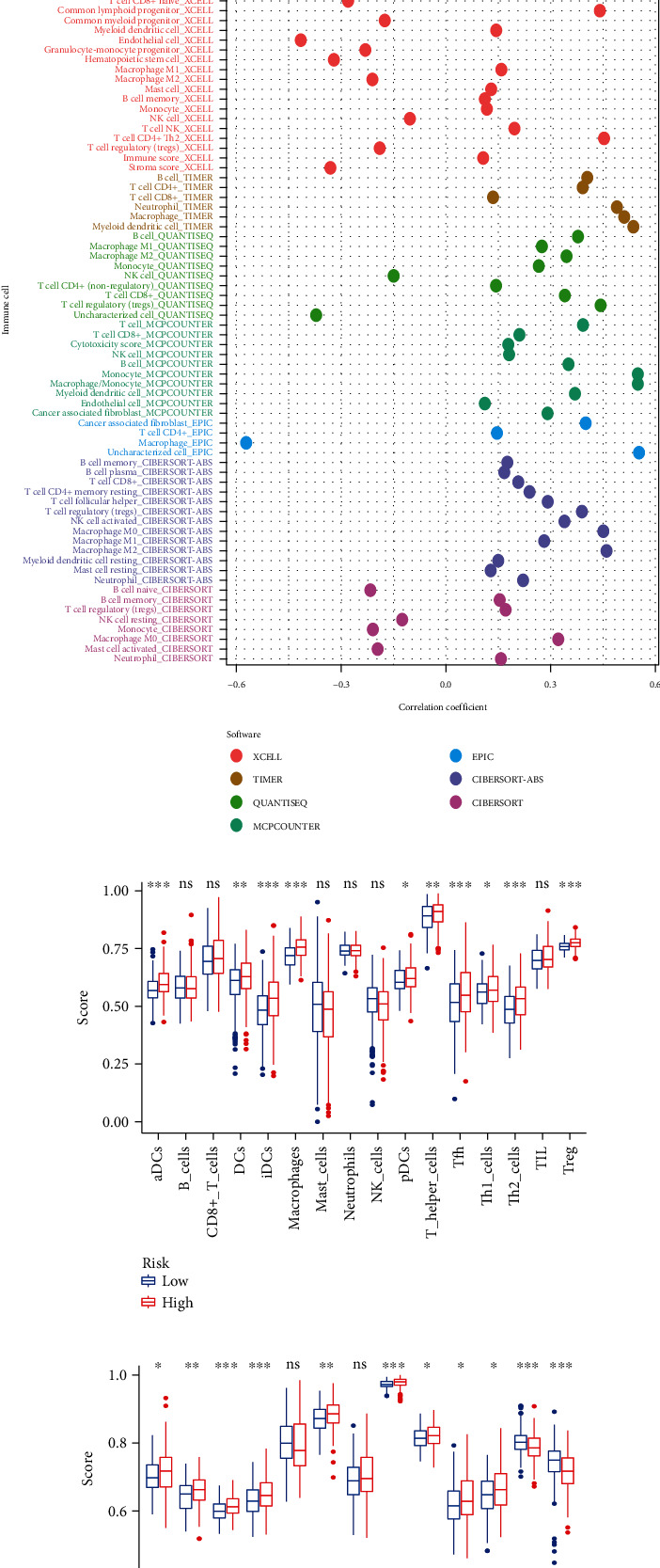
Immune infiltration analysis, correlation, and comparison of the high-risk and low-risk groups in TCGA. (a–c) The comparison of immune scores, stromal scores, and TIDE scores. (d) The correlation analysis of the immune infiltration and the risk scores. (e, f) The comparison of ssGSEA scores derived from 16 different immune cells and 13 immune signal pathways.

The infiltration of immune cells and stromal cells in HCC tissues were represented by the immune scores and the stromal scores, which were estimated by the ESTIMATE algorithm based on gene expression files of HCC samples in the TCGA-LIHC cohort. The Wilcoxon test indicated the stromal scores existed no significant difference between high-risk and low-risk groups, but the higher risk group tend to have higher immune scores, which suggested that the HCC in the high-risk group were more possible to have more abundant infiltration of immune cells (Figures 5(a) and 5(b)). The comparison of TIDE scores indicated the HCC in the high-risk group existed fewer dysfunctional and immune-excluded T cells (Figure 5(c)). The correlation analysis between risk score and the immune infiltration derived by various bioinformatic tools and algorithms was conducted, and most of the immune cells were positively correlated with the risk scores (Figure 5(d)). The ssGSEA scores of 16 different immune cells and 13 immune signal pathways for HCC samples with high and low risk were calculated and compared. Most of the immune-associated protein, cells, and activities were enriched in the high-risk group, like antigen-presenting cell (APC) coinhibition (*P* < 0.05), APC costimulation (*P* < 0.01), chemokine receptor (CCR, *P* < 0.001), checkpoint (*P* < 0.001), human leukocyte antigen (HLA, *P* < 0.05), major histocompatibility complex (MHC) class I (*P* < 0.001), parainflammation (*P* < 0.05), T cell coinhibition (*P* < 0.05), T cell costimulation (*P* < 0.05), aDCs (activated dendritic cell, *P* < 0.001), dendritic cell (DCs, *P* < 0.01), inhibited dendritic cell (iDCs, *P* < 0.001), macrophages (*P* < 0.001), plasmacytoid dendritic cells (pDCs, *P* < 0.05), T helper cells (*P* < 0.01), follicular helper T cell (Tfh, *P* < 0.001), Th1 cells (*P* < 0.05), Th2 cells (*P* < 0.001), and regulatory T cells (Treg, *P* < 0.001), but type I and type II interferon (IFN) responses (*P* < 0.001) were mainly enriched in the low-risk group (Figures 5(e) and 5(f)).

**Figure 6:**
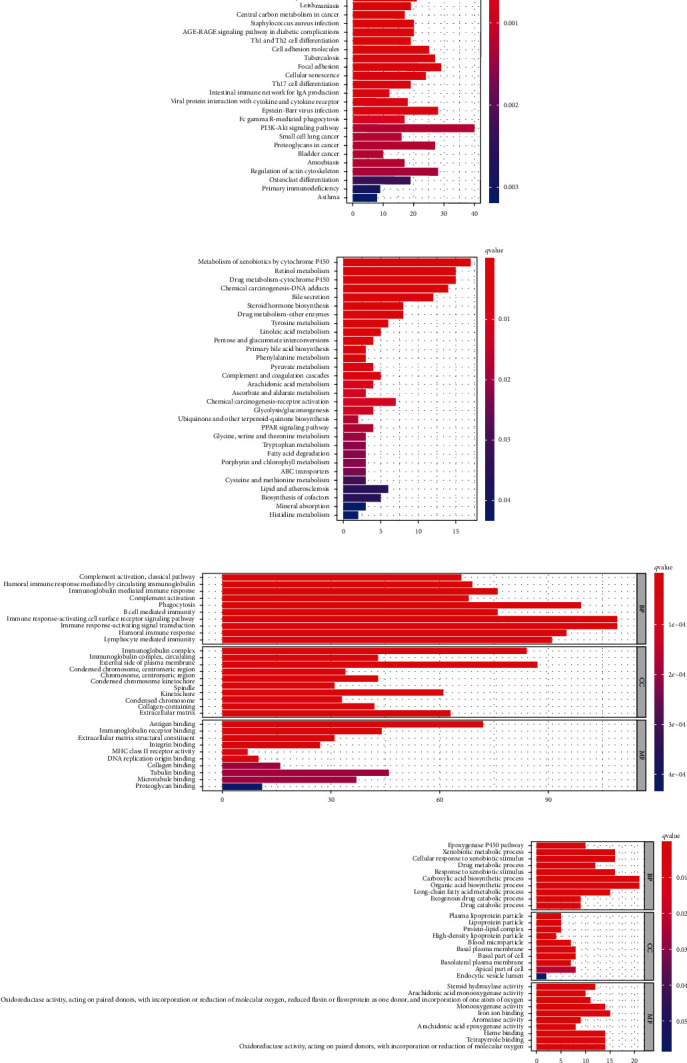
KEGG and GO enrichment analyses of the differentially expressed genes between high-risk and low-risk groups in TCGA. (a, b) The KEGG pathway enrichment analysis for the up/downregulated genes. (c, d) The GO term analysis, including biological process (BP), cellular component (CC), and molecular function (MF) for up/downregulated genes.

There were 1249 upregulated genes and 146 downregulated genes after the differential expression analysis. KEGG pathway gene enrichment analysis suggested the upregulated genes were mainly enriched in some functional pathways, including human T cell leukemia virus 1 infection, cell cycle, and phagosome (Figure 6(a)). GO term gene enrichment analysis indicated the upregulated genes were mainly enriched in immune-related biological pathways (immune response-activating cell surface receptor signaling pathway, immune response-activating signal transduction, and so on), cellular components (external side of the plasma membrane, immunoglobulin complex, and so on), and molecular functions (antigen binding, immunoglobulin receptor binding, and so on) (Figure 6(c)). The downregulated genes between high-risk and low-risk groups in TCGA-LIHC mainly enriched in the KEGG pathways, including metabolism of xenobiotics by cytochrome P450, retinol metabolism, and drug metabolism−cytochrome P450 (Figure 6(b)), metabolism-related biological pathways (carboxylic acid biosynthetic process, organic acid biosynthetic process, and so on), cellular components (basal plasma membrane, basal part of cell, and so on), and molecular functions (iron ion binding, monooxygenase activity, and so on) (Figure 6(d)). Therefore, the high-risk group might be more active in immune response rather than metabolism process when compared with the low-risk group.

**Figure 7:**
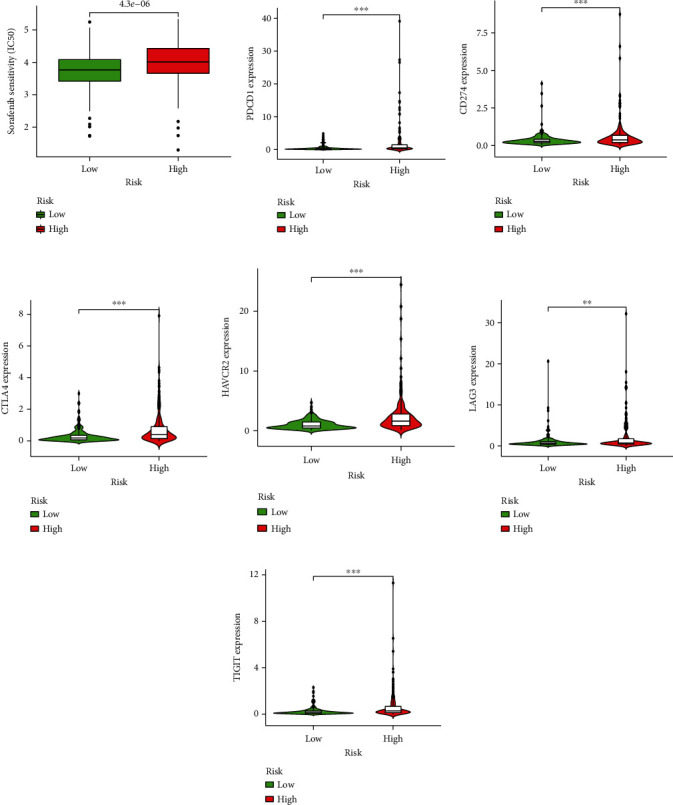
Drug sensitivity and immune checkpoint gene expression of high-risk and low-risk groups in TCGA. (a) Sorafenib IC50 comparison. (b–g) The immune checkpoint expression comparison, including PDCD1, CD274, CTLA4, HAVCR2, LAG3, and TIGIT (^∗∗∗^*P* < 0.001, ^∗∗^*P* < 0.01, and ^∗^*P* < 0.05).

The drug sensitivity of Sorafenib for HCC was evaluated via the pRRophetic package and compared by the Wilcoxon test. The boxplot exhibited that the low-risk group had a lower IC50 of Sorafenib than the high-risk group (Figure 7(a)), which suggested the low-risk group would be more sensitive to Sorafenib. The gene expression of immune checkpoints was compared and might predict the therapeutic efficacy of ICIs. The gene expression of PD-1, PD-L1, CTLA4, HAVCR2, LAG3, and TIGIT in the high-risk group was higher than the one in the low-risk group (all *P* < 0.05) (Figures 7(b)–7(g)), which indicated the high-risk group would have a higher response rate to the inhibitors for these immune checkpoints.

## 4. Discussion

**Figure 8:**
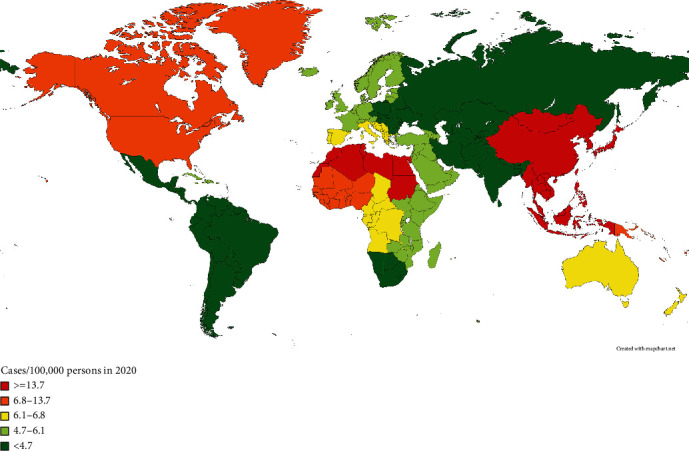
Geographic distribution of age-standardized incidence rates of liver cancer (reported as number of cases per 100,000 persons) was created with http://Mapchart.net using 2020 data from the World Health Organization (https://gco.iarc.fr/today/data/factsheets/cancers/11-Liver-fact-sheet.pdf).

As is shown in Figure 8, HCC is the prevalent hepatic malignancy worldwide, mainly distributed in Eastern Asia (17.8 cases per 100,000 persons), and poses a serious threat to human health [24, 25]. The comprehensive treatment, based on surgery and supplemented by interventional therapy, chemotherapy, and local therapy, has been widely used in the routine treatment and intervention of HCC. Radical surgical resection is the best option for HCC and can effectively improve the survival prognosis, but most patients are at an unresectable or advanced stage when diagnosed [26]. Nowadays, with the development of immunotherapy, especially ICIs, the systematic treatment of advanced HCC has made great progress, such as the first-line treatment combination of atezolizumab and bevacizumab [27]. However, the response rate of ICI plus antiangiogenic targeted therapy is approximately only 30% and limits the wide application in the clinic [28, 29]. Recently, ferroptosis, necroptosis, and pyroptosis have been found in tumor cell death and gradually emerged as a novel tumor treatment strategy in lots of studies [30]. Moreover, it is believed that the cell death mechanisms can synergistically enhance the antitumor immune activity [7].

Most of the pyroptosis-related genes were overexpressed in HCC and had an unfavorable effect on the overall survival prognosis, which indicated that the overexpression of pyroptosis-related genes was a prognostic factor of the poor outcome for HCC. The positive correlation among the genes suggested the genes might participate in the positive feedback or cascade reaction in HCC. The results of survival analysis revealed the survival outcome difference between the high-risk group and the low-risk group classified by the risk signature based on 6 pyroptosis-related genes. The distribution of risk scores and survival status further explained the risk stratification ability of the risk signature. The ROC curves further confirmed the considerable prediction ability of the risk signature. The results of PCA and *t*-NSE showed that the risk signature had a great clustering ability in the survival prognosis for HCC.

The higher expression of 6 genes incorporated into the risk signature predicted the worse overall survival outcome in HCC. BAK1, the gene encoding the BCL2 protein family, participates in the tumor apoptotic activity and the p53 signaling pathway. The overexpression of BAK1 is closely associated with the unsatisfactory survival outcome of HCC. BAK1 gene knockout can significantly inhibit proliferation and promote apoptosis of tumor cells in HCC [31]. CHMP4B is the subunit to consist of the endosomal sorting complex needed by transport-III complex and works in the cytokinetic membrane abscission and the mitotic cell division. The high expression of CHMP4B is related to poor survival prognosis and drug resistance to doxorubicin in HCC. Knockdown of CHMP4B can also restrict the cell proliferation of HCC [32]. DHX9 is an indispensable regulatory in transcription and translation, DNA replication, and maintenance of genomic stability, and the experiments in vivo and in vitro demonstrate that DHX9 suppression can conduce to tumor inhibition [33]. TREM2 is the triggering receptor expressed on myeloid cells, functions in immune response, and involves in chronic inflammation. TREM2 mRNA is highly expressed in HCC while its protein level is low. It is also the prognostic gene related to the tumor microenvironment. TREM2 can inhibit chronic inflammation and protect the liver from injury caused by some pathological changes, like hepatic fibrosis or cirrhosis, viral hepatitis, nonalcoholic fatty liver, and HCC [34]. GSDMC and DFNA5 (also known as GSDME) are the members of the gasdermin family. Our results suggest that high expression of GSDMC correlates with unfavorable prognosis, but GSDMC protein level is low in HCC according to the search results of the Human Protein Atlas, which might be related to the low value of transcript per million and the insufficient ability of immunohistochemical detection. Intriguingly, it is reported that nuclear PD-L1 translocation could promote the transcription of GSDMC and switches the apoptosis induced by TNF-*α* to pyroptosis in cancer, which further indicated the relationship between pyroptosis and immune checkpoint [35]. Active caspase-3 can process GSDME and produce N-terminal fragments to form pores in the cell membrane to induce pyroptotic cell death and activate the antitumor immune response, which means GSDME tends to be a cancer suppressor. Although the results indicate that the expression of GSDME in HCC is higher than that in normal tissues, the protein level is lower in HCC [36, 37]. Our results indicated the higher mRNA expression of GSDMC and GSDME is the indicator for the poor prognosis of HCC. The recent study also showed that the dysregulation of the gasdermin family might be related to the survival prognosis and immune infiltration of HCC [38].

The signature consisted of 6 pyroptosis-related genes that can successfully evaluate survival prognosis and conduct risk discrimination. The individual risk score is related to AFP level, tumor vascular invasion, and histological grade, and this also reflects the risk score is related to the tumor recurrence possibility. There was a significant positive correlation between the risk scores and the abundance of many types of immune cells. The high-risk group is more likely to have relatively higher immune cell infiltration and more active immune functions than the low-risk group, which further confirms the immune modulation ability of pyroptosis. The difference between the gene enrichment results of upregulated and downregulated genes may be related to immune response and metabolism process in HCC with different risk levels. The high-risk group is less sensitive to Sorafenib than the low-risk group but may be more sensitive to some immune checkpoints. It is reported that Sorafenib can regulate the crosstalk between natural killer (NK) cells and macrophages to execute the anticancer effect, especially inducing pyroptosis in macrophages and triggering NK cell-mediated cytotoxicity against HCC [39], which may be the reason why the low-risk group with less active pyroptosis state that could be activated is more sensitive to Sorafenib. Pyroptosis is the research direction that holds promise for the future, and some drugs that induce pyrolytic reaction have been reported [40].

This study still exists some limitations. Firstly, the functional profiles and molecular mechanism of 6 genes in HCC development and tumor immune microenvironment remain unknown and require further exploration in the future experimental studies. Secondly, due to the data limitation, the prediction and assessment performance of tumor survival outcome, recurrence risk, and drug therapy efficacy by the signature should be further verified by the relevant clinical trial data. Finally, the signature should be validated by the multicenter data and combined with more clinicopathological data to facilitate its implementation in the clinic.

## 5. Conclusions

In the present study, the pyroptosis-related genes were systematically analyzed and established and validated the risk signature on the basis of 6 differentially expressed genes with prognostic values (BAK1, CHMP4B, DHX9, GSDMC, GSDME, and TREM2). The risk signature can greatly predict survival prognosis and conduct risk discrimination. Clinicopathological feature comparison in different risk levels indicates that it may be related to tumor recurrence. The risk signature can somewhat predict the immune infiltration and the therapeutic efficiency for HCC. Six identified genes can be promising to become the pyroptosis biomarker and therapeutic targets of HCC.

## Data Availability

The data analyzed in this study could be accessible in the public databases. The gene expression profiles and the clinical data of the corresponding subjects were downloaded from TCGA-LIHC (http://cancergenome.nih.gov) and ICGC-LIRI-JP (https://dcc.icgc.org/) databases. The pyroptosis-related genes could be obtained in the dataset and the literature mentioned in methods. The data were analyzed in R software version 4.1.0 (https://www.r-project.org/). The R codes and the processing data could be obtained by contacting the authors.
